# The Youth Risk Index: psychometrics, predicting the initiation of early adolescent substance use, and the breadth of liability detected

**DOI:** 10.3389/frcha.2025.1513607

**Published:** 2025-06-27

**Authors:** Ty A. Ridenour, Nisha O'Shea Gottfredson, Jason Williams, Daniel S. Shaw, Maureen D. Reynolds, Cheryl A. Roberts, Richard Spoth, David R. Garnica-Agudelo, Idil Baran, Aysenil Belger, Diana H. Fishbein

**Affiliations:** ^1^ Substance Use Prevention, Evaluation, and Research, RTI International, Research Triangle Park, NC, United States; ^2^ Department of Pharmaceutical Sciences, University of Pittsburgh, Pittsburgh, PA, United States; ^3^ Frank Porter Graham Child Development Institute, University of North Carolina, Chapel Hill, Chapel Hill, NC, United States; ^4^ Partnerships in Prevention Science Institute, Iowa State University, Ames, IA, United States

**Keywords:** conduct problems, screening, disinhibition, social contagion, validity, substance use and misuse, substance use disorder liability

## Abstract

**Introduction:**

Over the last two decades, drug use epidemics have occurred across the world, including in countries with well-funded services for treatment and recovery, underscoring the need to bolster primary prevention. Moreover, substance use (SU) and SU disorders (SUDs) contribute to the etiology and exacerbation of many medical and psychiatric illnesses. The first step in providing selective/indicated prevention for SU/SUD is identifying high liability (overall risk). We evaluated the *Youth Risk Index*© (YRI) screening tool, which measures liability to SU and related behaviors, frequently before they are initiated, at ages 8–14.

**Methods:**

Using data from five previously recruited samples of youth, psychometric analyses consisted of (1) confirmatory factor analyses comparing two latent structures, (2) non-invariance tests between sexes and purposes for using the YRI (research or screening), and (3) concurrent and predictive validity. Reports from 4,495 youths aged 8–13 were analyzed, with approximately half of the sample representing each sex, each research purpose, and a Caucasian identity.

**Results:**

A latent structure with one second-order factor (Overall Liability) and three first-order factors (Disinhibition, Peer Conduct Problems, and Social Contagion) best fit the observed data and was well-replicated within sexes and purposes. Partial scalar non-invariance between purposes occurred for Overall Liability involving two items. Disinhibition had partial non-invariance between sexes and purposes involving the same item. Greater non-invariance was found for Peer Conduct Problems and Social Contagion. Traditional and non-invariance-adjusted scores correlated highly, with values of 0.96 for Overall Liability, 0.99 for Disinhibition, 0.89 for Peer Conduct Problems, and 0.93 for Social Contagion. Traditional scoring provided a good to excellent area under the receiving operating characteristic curve for concurrent and 1-year SU and conduct problems. Greater YRI scores were associated with greater youth-reported depression, sensation seeking, substance use, conduct disorder behaviors, and parental problems from SU and/or legal problems, as well as less self-management and parent fortification and poorer performances in planning, problem-solving, and low-load working memory tasks. YRI scores correlated less with parent reports on youths than with youth self-reports.

**Discussion:**

In sum, YRI scores encapsulate many segments of risk for SU/SUD and related behavior problems, which is critical to accurately identify the need for and provision of selective/indicated prevention because of the manifold risk factors for, and complex etiology of, SU/SUD.

## 1 Introduction

Over the last decade, multiple drug use epidemics have impacted disparate regions of the world, such as psychostimulants in Europe and the Middle East, opioids in North America and Asia, and inhalants in South and Central America, with consequential or associated epidemics in mental health or drug overdose and societal burdens such as crime and medical illness (1–4). Substance use (SU) specifically contributes to the risk for and exacerbation of many medical and psychiatric illnesses (5–8). The medical, mental health, and societal burdens from SU continue even in countries where large investments have been made to expand drug treatment, recovery, and overdose prevention (9, 10), underscoring the need to bolster primary prevention.

Although adolescent SU has decreased over the last 30 years, the burden of disease from adolescent SU has increased and continues to greatly outstrip the resources that are dedicated to prevention and treatment. From 1990 to 2019, alcohol and drug use prevalence decreased on average in 31 European countries among those between the ages of 10 and 19 (11). For example, the prevalence of adolescent past-month alcohol use has declined since 1995 on average across 39 higher-income countries, even though the magnitude of change varied considerably among nations (from increased use to an 83.9% decrease) (12). In contrast, the years lived with a disability due to drug use increased by 17.7% among Europeans aged 15–24 (11). Evaluating the change in Global Burden of Disease due to adolescent substance use disorders (SUDs) over roughly the same period, Yu and Chen (13) demonstrated a growing international need for the prevention and treatment of adolescent SUDs, especially in higher-income countries and where the prevalence of adolescent SUDs is already high. Moreover, the relative dearth of evidence on SU among children and adolescents who reside outside of these nations illustrates the critical need for research on the international etiology of, early detection of risk for, and prevention of SUDs.

Recent US trends demonstrate how the burden of disease increased in this country despite its overall decrease in adolescent SU. The burden has grown exponentially over the last 10 years, specifically due to the misuse of opioids, stimulants, and fentanyl, along with the latter’s derivatives and lacing, and the resultant overdoses and deaths (14, 15). These trends occurred despite large investments to expand drug treatment, recovery, and overdose prevention (9, 10). In contrast, funding to prevent early adolescent SU was reduced, which presages the misuse of high-potency life-threatening drugs, drug overdoses (that disproportionately impact teens and young adults), SUDs, and other developmental “snags” that contribute to SU-related problems, including poor school attendance and performance, conduct problems, and unsafe sex (16–21). Moreover, early adolescent SU is common in the US; in 2022, the prevalence of lifetime SU among 8th graders was 23.1% for alcohol, 18.1% for vaping, 11.0% for marijuana, and 32.9% for any SU.

With the goal of developing prevention specifically for children and early adolescents with a high risk for SU-related problems, this study expanded prior research to more rigorously evaluate an innovative tool to detect high risk for SU and related behaviors among those aged 9–14. Screening with this tool could, in turn, allow youths with increased vulnerability to receive selective/targeted prevention, frequently years before problematic SU might otherwise occur.

The instruments that have most closely measured high risk for SU-related problems prior to SU initiation has been tools used to measure specific SU risk factors to evaluate prevention programs (22). Historically, SU/SUD measurement has emphasized forms of use (e.g., to track prevalence and trends), international development of diagnosis and nomenclature tools, and use-related pathology (23, 24). In the US alone, three nationally representative, annual, and decades-long surveys monitor adolescent SU patterns (25–27). Perhaps a testament to the severity of SU harm, burden to society, resistance to treatment, and complexity in etiology, thousands of instruments have been developed to measure features of SU, its risk factors, clinical outcomes, and its effects (28–31).

The *Youth Risk Index*^©^ [YRI (32)] was created to detect high risk for SU in late childhood and early adolescence before or soon after SU is initiated to facilitate selective/indicated prevention for these youths (33–35). The YRI is based on the liability-threshold model, the preeminent transtheoretical model of SUD etiology (36, 37). The liability-threshold model states that the overall risk (liability) for SU and SUD is normally distributed due to manifold biopsychosocial factors that can sway individuals’ development toward or away from SU (38). Once an individual's liability surpasses a hypothetical threshold, the corresponding level of SU or SU-related problems occurs. The most pertinent SU thresholds during early adolescence correspond to the initiation and escalation of use (e.g., regular or binge SU), and less prevalent SUDs.

The YRI's 23 items were selected from the *Assessment of Liability and Exposure to Substance use and Antisocial behavior, Revised*^©^ (ALEXSA-R^©^) system based on how well they predict SU or conduct problems (defined as two or more conduct disorder behaviors) up to 1 year later. The ALEXSA-R is a youth-reported psychological assessment system composed of well-validated measures of SU vulnerabilities and resiliencies, early clinical forms of SU, externalizing behaviors, and internalizing symptoms for youth aged 8–14 (34, 35, 39). It is an illustrated computer-administered self-interview platform with audio, and its choreographed presentation of stimuli allows it to be completed by youth with illiteracy or other reading difficulties. Scores include standardized norms from a US nationally representative sample. ALEXSA-R subscales are organized into 10 domains of risk that were identified in factor analyses (Disinhibition, Family Discord, Parental Fortification, Risk Behavior Perceptions, School Protection, Self-management, Sensation Seeking, Social Contagion, Social Support, and Neighborhood Risks), or as “stand-alone” subscales (e.g., boredom, pubertal status, and religiosity). Its clinical measures include Aggression, Anxiety, Conduct Disorder Behaviors, Delinquency, Depression, Suicidal Ideation, and SU Risk Indexes.

The YRI items were selected from the ALEXSA-R vulnerability and resiliency subscales (collectively “risk factors”) using longitudinal data from 640 youths, aged 9–13, who were experiencing chronic stress. Item selection was based on how well it predicted either (1) co-occurring SU (tobacco, alcohol, and/or cannabis) or having at least two conduct disorder behaviors (termed conduct problems), or (2) experiencing these behaviors during the subsequent year (32, 35). The YRI's overall score predicted SU and/or conduct problems with large odds ratios. The YRI screening tool does not include questions about SU or other potentially stigmatizing topic (a priority for many healthcare providers); can be administered by behavioral specialists or nurses; has an 8-min administration time; does not disrupt the “flow” of a Well-child Check-up; and has ratings of almost universal acceptability to patients, parents, and pediatric staff (32, 33). The YRI is traditionally scored using the mean of the raw item scores with a possible range of 0–3.5.

For screening purposes, two YRI score thresholds identify the low-, moderate-, and high-risk ranges. Scores above the high-risk threshold (1.55) have 80% sensitivity for SU and/or conduct problems, while scores below the moderate-risk threshold (0.87) have 80% specificity for not experiencing SU and/or conduct problems (32). In two samples of patients recruited during well-child office check-ups, the YRI was evaluated as a screening tool to detect youths with moderate to high risk for SU. In the first sample, high-risk youths and their parents were referred to family counseling (32). In the second sample, they were invited to enter a clinical trial in the *Family Check-Up* prevention program (32, 33). Near universal approval of the screening and referral protocol was reported by parents, youths, and pediatric staff, in terms of its overall acceptability (96%, 89%, and 100%, respectively), the importance of pediatricians helping youths to behave safely (96%, 93%, and 100%, respectively), and specific features of the screening protocol. All pediatricians reported that the screening and referral protocol did not impede patient “flow.” Of the parents who were offered the *Family Check-Up* prevention program, 93.5% enrolled in and completed it; exposure to the program was associated with youths having less SU, less initiation of new substances, less anxiety, and other lower vulnerabilities (33).

**Figure 1:**
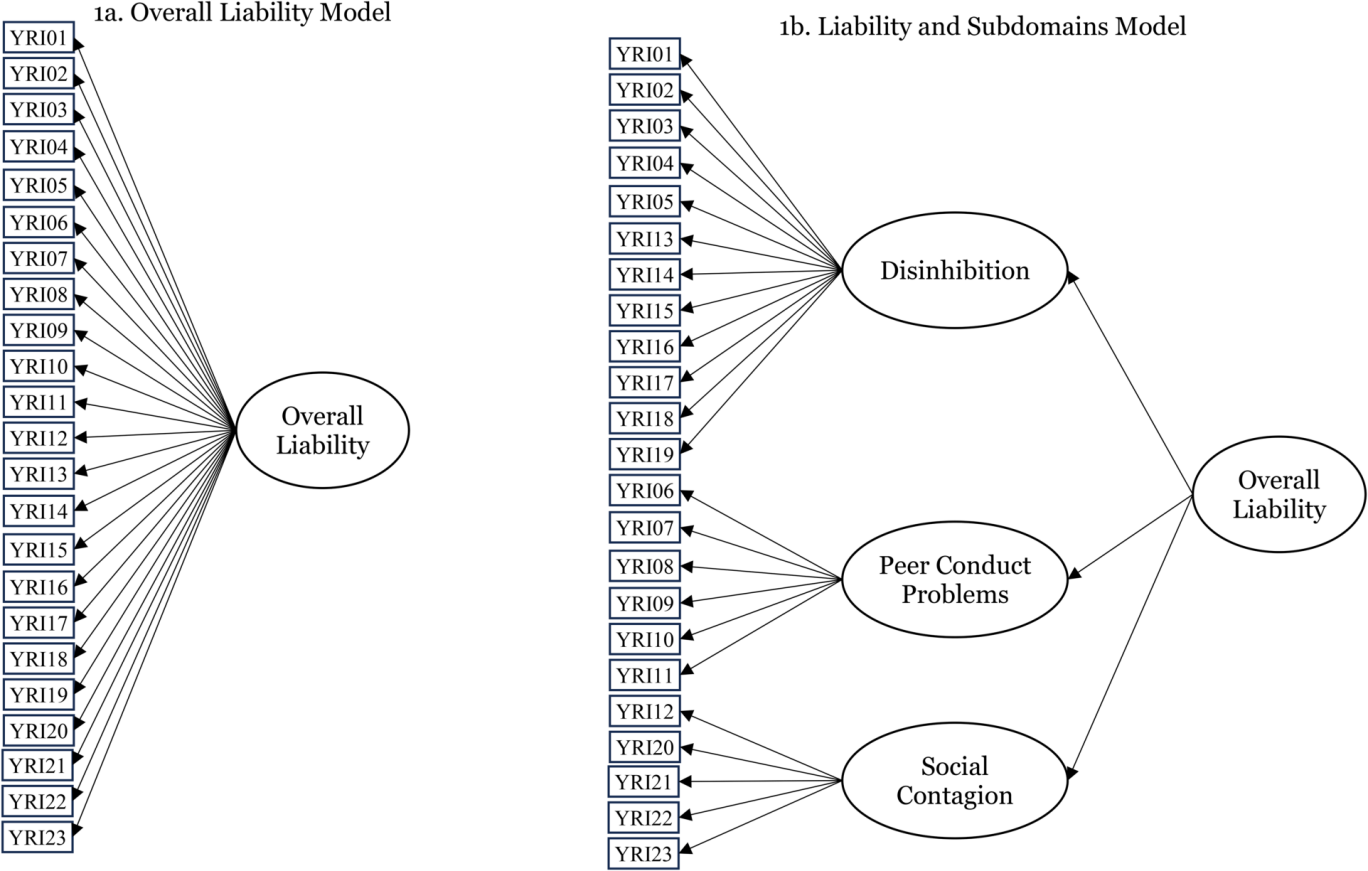
Competing latent structures for the *Youth Risk Index* measurement model. (**a**) Overall Liability model. (**b**) Liability and Subdomains model.

In the present study, we evaluated three assumptions underlying the YRI's measurement model and scoring. First, we tested the assumed YRI latent structure, which has traditionally consisted of a single latent variable onto which all YRI items load (Figure 1a). This Overall Liability model was based on the liability-threshold model and the fact that all the items were selected because they statistically predicted common outcomes (SU and/or conduct problems). However, an alternate latent structure could also be consistent with the liability-threshold model, potentially providing a better fit of the observed data and yielding more complete information for providers. This alternate Liability and Subdomains model adds three first-order factors consisting of the ALEXSA-R domains of risk from which the YRI items were drawn (Disinhibition, Peer Conduct Problems, and Social Contagion) (Figure 1b). All the item loadings onto their corresponding first-order factor were hypothesized to exceed 0.40, and the first-order factor loadings onto liability were hypothesized to be large (i.e., at least 0.60).

To evaluate the need for a more nuanced latent structure, we tested measurement invariance [i.e., no differential item functioning (DIF)] of YRI items across sex (youths reporting to be a boy or a girl) and the purpose for which the YRI was completed (research study vs. intervention screening). If non-invariance was found, it was hypothesized to be limited to a few items (partial non-invariance) that could be resolved in future research. Measurement invariance testing progressed from general to more specified models (40). Configural invariance indicates that the general latent structure (number of factors and significant item loadings) does not differ across groups. Metric invariance requires equivalent factor loadings across groups. Scalar invariance requires equivalent factor loadings and intercepts across groups. Partial non-invariance was indicated when specific items could be identified that were the source of metric or scalar non-invariance so that subsequent adaptations to the YRI score could account for the non-invariance.

Second, we hypothesized that the best-fitting YRI measurement model has concurrent and predictive validity in the form of large odds ratios and area under the receiving operating characteristic (ROC) curve for SU (concurrent and at 12-month follow-up), conduct problems (concurrent and at 12-month follow-up), and a breadth of risk factors. Moreover, concurrent and predictive validity were hypothesized for scores that were and were not adjusted for any differential item functioning that was found.

Our third hypothesis was that the YRI scores would account for variation in certain dimensions of neurocognitive functioning (and their corresponding neural substrates) that have previously been found to predict SU and SUDs in adolescents (41, 42). Specifically, cognitive and neuroimaging studies have shown that working memory (43), impulse control (43, 44), and emotion regulation (45–48) are potent predictors of initiation and subsequent escalation of SU in adolescents. Relative deficits in these functions have also been identified in children and adolescents with behavioral problems known to antedate SUD, such as attention deficit hyperactivity disorder [ADHD (49)], hedonia coupled with low cognitive control (50), and other externalizing behaviors (51, 52), further illuminating the potential developmental and behavioral pathways to SUDs. Delineating the association between the YRI and the neurocognitive functions found to predict adolescent SUD would be consistent with the YRI's validity in discriminating among individuals’ levels of SU liability, including “under the skin” factors. The biological underpinnings of the liability-threshold model and the corresponding large body of literature attest to the sizable neurobiological, genetic, physiological, and cognitive contributions to SUD liability, which YRI screening could detect without the need for invasive or extensive testing.

## 2 Materials and methods

All study methods, recruitment and consenting, confidentiality protections, and data storage procedures followed the reviews and approvals of IRBs at the institutions where the original studies were conducted. The datasets selected for our secondary analyses were chosen to afford us large subsamples of each sex and of two purposes for administering YRI (either research or clinical screening), which were required to evaluate the latent structure of the YRI and to sufficiently test for measurement equivalence. Collectively, the studies represented diverse study designs: convenience community sampling, elevated risk sampling (for SUD), and a clinical trial. Participants were not randomly selected from the population of US youths; rather, they were recruited for the original studies’ purposes, which are described below. The samples were also selected partly because of the range of validity criteria that they provided, spanning youth reports, parent reports, and cognitive tasks with known neurophysiological underpinnings that are associated with SUD.

### 2.1 Samples

Data from five previously recruited samples of youth who completed the YRI were analyzed (Table 1). Four of these samples, the “Research Samples” and “Screening Samples,” collectively offered nearly equivalent numbers of participants of both sexes and of youths who completed the YRI for research purposes or screening for intervention. No data were collected from the Sixth Graders Sample other than the YRI. Hence, the tests of the YRI's concurrent and predictive validity in association with well-known SU/SUD risk factors included data from only three of these samples. The fifth sample's data specifically tested YRI validity compared to neurocognitive functioning.

**Table 1:** Sample demographics.

Sample characteristic	Research samples		Screening samples		Aggregated invariance sample	Validity only
	Standardization	Chronic Stress	Sixth-grade Students^a^	Well-child Check-up		CogNIT
Sample size	641	1,535	1,661	625	4,462	33
Sex (% female):	49.8	50.0	48.4	52.0	49.7	39.4
Age ($\bar{X}$, SD):	10.6 (1.69)	10.5 (1.48)	—	11.3* (1.17)	10.7^b^ (1.50)	13.7 (2.01)
Race/ethnicity (%)^c^						
African American	14.9%	11.6%	—	77.8%*	27.1%^b^	30.3
American Indian	0.8	0.9	—	2.2	1.2^b^	0.0
Asian American	2.7	0.9	—	0.7	1.3^b^	0.0
Caucasian	45.3	60.7*	—	11.0	46.1^b^	60.6
Latin American	9.6	4.0	—	7.2	6.0^b^	9.1
Mixed	23.3	18.0	—	12.6	18.0^b^	3.0
Other	3.4	3.9	—	2.9	3.6^b^	0.0
YRI score ($\bar{X}$, SD)^d^	0.88 (0.47)	1.13* (0.58)	0.85 (0.47)	1.07* (0.56)	0.98 (0.54)	2.22 (1.56)
YRI alpha	0.86	0.87	0.87	0.87	0.87	—

Alpha = Cronbach's alpha estimate of internal consistency. Cell values within a demographic may not sum to 100 due to rounding error. Respondents could have selected “Do Not Know,” “Refuse to Answer,” or simply not responded to the question; those who did not provide either “male” or “female” responses were not included in the gender invariance analyses in part due to the small subsample size. Cell value was greater than samples that are not denoted with * at *p* < 0.001 (*χ*^2^ = 122.72 for African Americans, 54.42 for Caucasians; Dunnett T3 multiple comparisons indicated greater YRI means for the Chronic Stress and Well-child Check-up samples compared to the other two).

^a^ School classroom screening was conducted without explicitly collecting age or other demographics.

^b^ Does not include the Sixth-grade Students sample because race/ethnicity was not collected in that sample.

^c^ Race/ethnicity percentages that do not sum to 100% are due to rounding error.

^d^ YRI score computed using the traditional mean of item scores.

#### 2.1.1 Chronic Stress sample

Between 2004 and 2010, 9- to 13-year-olds (*N* = 1,535) attended a summer camp in the northeastern US that was designed for youths experiencing chronic stress. Based on an adult sponsor letter and interview with a school staff member who knew the youth, camp staff categorized sources of a youth's chronic stress as family poverty, serious family problems (e.g., incarcerated parent), social problems (e.g., severe peer rejection), poor academic performance, or emotional problems (e.g., mood disorder but diagnoses were not made during the study), but did so without using standardized tools. Camp staff ratings suggested that the youths had a mean of 2.4 (SD = 1.3) of these stressors. The participants were from urban, suburban, and rural residences (the data for which were not quantified). The youths completed the YRI and other ALEXSA-R subscales while attending camp as part of the program evaluations. Although many youths attended the camp over multiple years, the data analyzed here were only from the youths' first year of camp attendance. More details regarding camp eligibility, data collection, and longitudinal findings are reported in previous studies (35, 39, 53–55). Chronic Stress sample data were analyzed to evaluate the YRI's latent structure and validity.

#### 2.1.2 Sixth Grader sample

Sixth-grade students (*N* = 1,661) completed the YRI to screen for their eligibility to receive preventive intervention from 2017 to 2021. They were from rural, suburban, and urban public schools across a Midwestern state. Parents of students with moderate or high risk were offered a brief preventive intervention and referral to additional behavioral health services for the student at no cost to the family. Sex was the only variable collected other than YRI screening. The YRI was group-administered to students in classrooms using tablets and website-based surveys. Student responses and YRI scores were stored remotely to protect student confidentiality, and parents were notified by mail of their eligibility to receive the preventive intervention. The Sixth Grader Sample data were included only in the analyses of the YRI's latent structure.

#### 2.1.3 Standardization sample

From 2014 to 2015, 641 youths, aged 8–13, were recruited from contiguous US regions other than the Northeast to derive standard scores that were normalized to the US population (standardization analyses included data from previously recruited participants from the Northeast). Participants were recruited from areas around Atlanta, GA; Boston, MA; Chicago, IL; Dallas, TX; Durham, NC; Fort Smith, AR; Los Angeles, CA; San Francisco, CA; and Washington, DC. Participants were recruited by private, for-profit survey companies from their pool of parents and youths who were interested in participating in research. Parents responded to advertisements for the study sent out by the survey companies to their pool of pre-registered, eligible participants. The sole exclusion criterion for youths (in addition to age) was having a severe developmental delay. Standardization Sample data were used in the analyses of the YRI's latent structure and concurrent validity.

#### 2.1.4 Well-child Check-up sample

From 2012 to 2018, two subsamples of patients attending Well-child Check-ups were recruited from the same healthcare system to in part evaluate the acceptability and feasibility of using the YRI for screening within primary care (32, 33). The first subsample of 61 patients, aged 9–12, was recruited at a suburban pediatric private practice (72.2%) or a rural hospital (27.8%) to evaluate the feasibility of the screening protocol. Youths and parents were consented and screened by nurses while they waited for physicians in an examination room. Inclusion criteria required participants to be English-speaking and not have a moderate or severe intellectual disability (per patient records). The parents of youths who scored in the moderate- or high-risk range were provided with contact information for a family therapist who had agreed to work with families to reduce the youths’ risk. This subsample's data were included in the analyses of the YRI's latent structure and concurrent validity.

**Figure 2:**
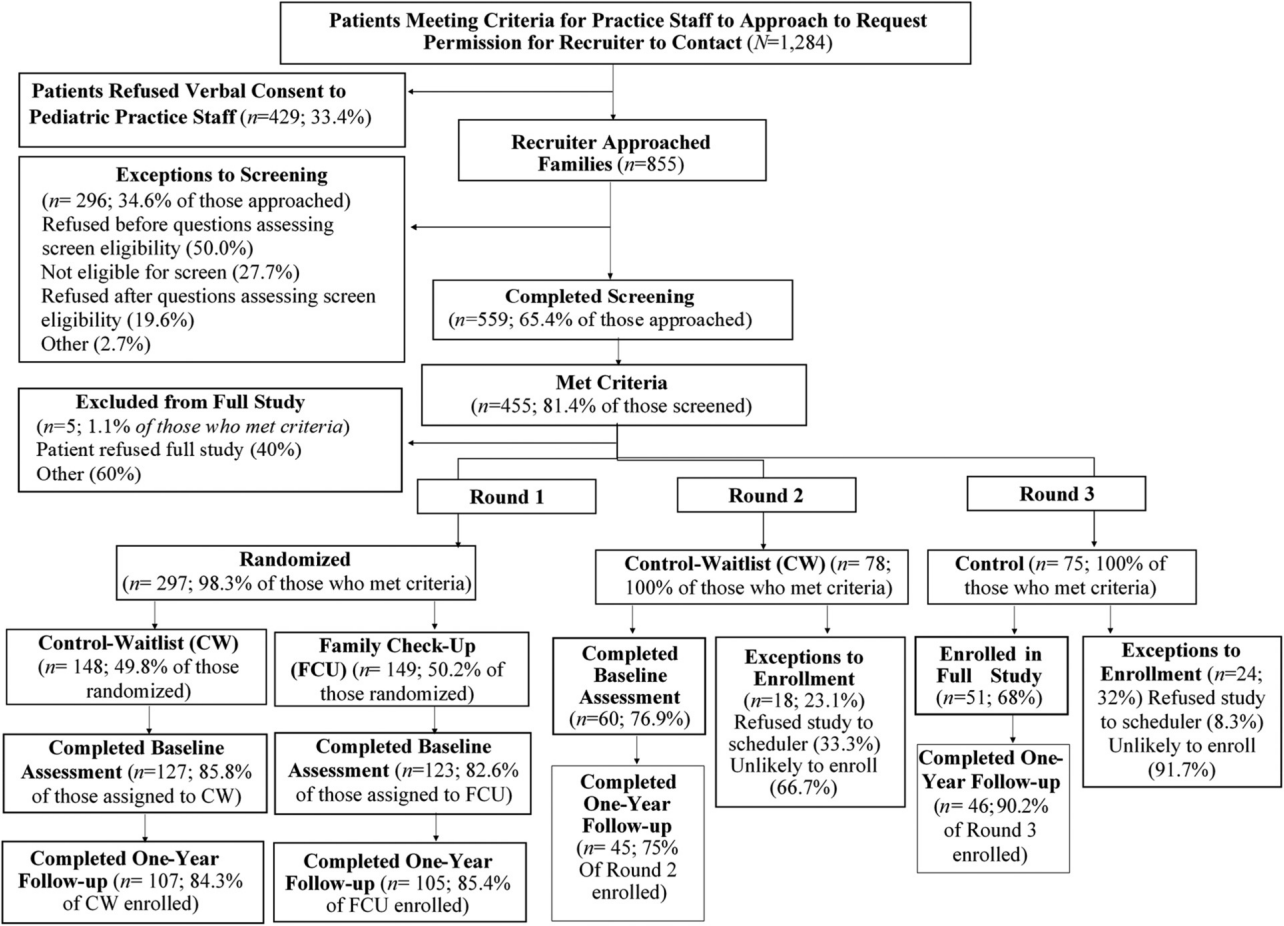
CONSORT diagram for the second Well-child Check-up sample (33).

The second well-child subsample of 564 patients, aged 10–13, was recruited for a clinical trial at a hospital-based pediatric practice that served patients residing in low-resource, urban neighborhoods (33). After the pediatric staff obtained verbal consent for research contact, the study staff approached families in exam rooms to obtain parent and youth assent and conduct YRI screening. If a youth's (or parent's) screening score was in the elevated risk range, the family was invited to participate in a clinical trial in the *Family Check-Up* prevention program. The other inclusion criterion was that the child had received needs-based Medicaid or the family income was at or below 150% of poverty guidelines. Exclusion criteria included an inability to speak English or the child having a moderate or severe intellectual disability. Data from this sample were included in the latent structure and validity analyses. The CONSORT diagram for the participant recruitment process is presented in Figure 2.

#### 2.1.5 CogNIT sample

This community sample of 60 youths, aged 9–18, participated in a neurodevelopmental study on the impacts of stress and trait anxiety on the maturation of neural circuitry that underlies stress regulation, emotion, and reward processing. A subsample of 33 participants completed the YRI, which was added to the protocol after the study was underway. Inclusion criteria were a higher-than-average level of stress, increased risk of psychosis, and a familial background of schizophrenia or bipolar disorder. Participant data were analyzed to evaluate the YRI’s validity for capturing the neurocognitive risk of SU/SUD.

### 2.2 Instrumentation

The YRI consists of 23 items from the ALEXSA-R^©^ assessment system (34, 35) that most accurately predicted the use of alcohol, tobacco, and/or cannabis or having 2+ conduct disorder behaviors 1 year later in 9- to 12-year-olds (Table 2) (32). The YRI items were from subscales that measure anger coping, distractibility, impulsivity, friends’ conduct problems, tobacco accessibility, social disinhibition, and peer pressure susceptibility. Five items were recoded for our analyses based on their raw score distribution departures from skewness = 0 and kurtosis = 3 so that they better aligned with the assumed underlying normal distribution (56). For the psychometric analyses, all the YRI items were handled as ordinal variables.

**Table 2:** *Youth Risk Index* item characteristics.

Item	*N*	Mean	SD	Skewness	Kurtosis	Notes	Item description
YRI01	4,155	2.13	0.82	0.70	0.22		Gets mad at people
YRI02^a^	4,157	1.47	0.74	1.70	2.62	Trichotomized	Takes frustration out on someone else
YRI03	4,170	1.30	0.58	2.26	5.73		When upset, criticizes or blames other people
YRI04	4,172	1.60	0.78	1.31	1.37		When upset, yells or screams at someone
YRI05	4,076	1.58	0.86	1.46	1.31		When upset, does something exciting or risky
YRI06	2,468	1.47	1.83	0.92	−0.64	Count	Friends have broken curfew
YRI07	2,647	0.76	1.41	1.96	2.78	Count	Friends have skipped school
YRI08	2,716	1.43	1.70	1.04	−0.22	Count	Friends have started fights
YRI09	3,742	0.86	1.51	1.79	1.96	Count	Friends have scared someone to get them to do what they wanted
YRI10^a^	3,817	0.26	0.90	4.11	16.85	Dichotomized	Friends have trespassed
YRI11	3,688	0.62	1.31	2.35	4.55	Count	Friends have vandalized property
YRI12^a^	3,673	0.42	0.88	2.05	2.91	Dichotomized	Level of access to tobacco
YRI13	4,115	0.57	0.85	1.51	1.52		Bothers other students when they are trying to work
YRI14	4,150	1.26	1.07	0.44	−1.05		Easily distracted from schoolwork
YRI15	4,089	0.84	0.95	1.01	0.07		Gets frustrated over difficult tasks and quits
YRI16	4,084	1.30	1.05	0.41	−1.00		Has trouble staying focused
YRI17	4,015	1.00	1.01	0.79	−0.46		Does things without stopping to think
YRI18	3,965	0.95	0.97	0.85	−0.23		Gets involved in things that later regrets
YRI19	4,016	0.85	0.94	0.96	0.04		Gets into trouble for doing things without thinking
YRI20^b^	2,284	0.69	0.96	1.30	0.63		How much do you like movies with lots of kissing in them?
YRI21^a^	4,274	0.12	0.47	4.47	21.37	Dichotomized	Would tear a page out of a library book on a dare
YRI22	4,184	0.77	0.93	1.09	0.26		Would watch a movie with a friend when needing to study for a test
YRI23^a^	4,274	0.07	0.39	6.27	40.61	Dichotomized	Would smoke with a friend even if parents were against it

Curran et al. (56) and others have suggested that departure from normality is indicated by skewness outside of −2 to 2 or kurtosis outside of *−*4 to 10 (compared to normal distribution with skewness = 0 and kurtosis = 3). Respondents could have selected “Do Not Know,” “Refuse to Answer,” or simply not responded to the question; for the analysis of YRI items, these responses were recoded as missing.

*N*, number of respondents; SD, standard deviation.

^a^ Item was recoded for scoring and analyses due to departure from normality.

^b^ Item was omitted from the sixth-grade students' screening.

#### 2.2.1 Youth reports

Youth reports of the risk factors that were used for the validity analyses were collected in the Chronic Stress, Standardization, and Well-child Check-up samples using other measures from the ALEXSA-R. The ALEXSA-R is an illustration-based computer-assisted self-interview with audio that measures risk factors for, and early manifestations of, youth SU and problem behaviors. Its items are organized into domain scores and individual subscales (based on factor analyses), which are reliable and valid for youth ages 7–19 of different races, genders, and literacy levels (34, 57). Self-management quantifies learnable skills that reduce the probability and severity of harmful consequences from mistakes, emotional dysregulation, or behavioral disinhibition, including SU, using three subscales: Concentration, Planning, and Problem Solving (54). The Sensation Seeking domain is based on Zuckerman's theory and research, using measures that were adapted for late childhood and early adolescence: Gambling, Social Disinhibition, and Thrill Seeking (34, 58). Parent Fortification is composed of three subscales that quantify facets of parent–offspring relationships that are negatively associated with SU/SUD: Parental Monitoring, Parental Attachment, and Parental Nurturance (34). The School Protection domain's four subscales quantify aspects of youths’ academic performance and school-based relationships that are negatively associated with SU/SUD: Academic Competency, School Bonding, School Commitment, and School Atmosphere: Adults (the last measures a youth's perception of how supportive school staff are of students) (34). Some individual ALEXSA-R subscales were also analyzed: Depression, Count of Substances Used, Conduct Disorder Behaviors (a count of behaviors), Parental Problems with the Law (binary), and Parental Problems from SU (binary) (34).

#### 2.2.2 Parent reports

Parent participants in the larger Well-child Check-up subsample completed well-established and widely used instruments. While useful for testing whether parent reports are associated with YRI scores, we expected small correlations at best, based on (1) the well-replicated finding that parent and youth reports on youth characteristics are largely discrepant (59) and (2) parents reported on different characteristics than those that are measured by the YRI items. The *Child Behavior Checklist* subscales of internalizing symptoms and externalizing behaviors were hypothesized to correlate positively with YRI scores (60). The three parent report measures, which are included in the *Family Check-Up* assessment battery, were Parental Monitoring, Parent Supports Good Behavior, and Parental Rule Setting (61).

#### 2.2.3 CogNIT measures

To estimate the degree to which YRI scores reflect cognitive functioning and underlying neural substrates that contribute to SU/SUD liability, a subset of the CogNIT study measures was analyzed. The *Wechsler Abbreviated Scale of Intelligence*'s (WASI) Forward Digit Span measures attention deficits. The subscales of the *Delis–Kaplan Executive Function System* (D-KEFS) included the Planning-sequencing Tower Test [quantifying planning and problem-solving abilities (62)] and the Color-Word Interference Test [processing speed and executive functioning (63)]. A fractal (N-back) test quantified working memory (64, 65, 94). The CogNIT participants completed the YRI in person, along with the neurocognitive tests, prior to MRI or EEG recordings.

### 2.3 Analyses

The descriptive summaries of samples (Table 1) included analyses of variance with Dunnett T3 multiple comparisons tests because the sample variances were heterogeneous. Data from 14 participants were removed because their ages were outside the 8–13 age range. Cases in which age was not reported (*n* = 1,681, almost entirely from the Sixth Graders Sample) were assumed to fall within the 8–13 age range. Data were managed in R Studio and then analyzed using Mplus (66).

#### 2.3.1 YRI item distributions

Most of the YRI items are ordinal, four-option Likert items, whereas YRI06–YRI11 are six-option count variables. Several YRI items were right-skewed and some were extremely zero-inflated, including YRI02 (takes out frustrations on others), YRI04 (yells at someone), YRI08 (friends start fights), YRI09 (friends bully others), YRI10 (friends have trespassed), YRI11 (friends have vandalized), YRI12 (access to tobacco), YRI13 (bothers students who are working), YRI21 (minor illegal dare), and YRI23 (would smoke). Two items had bimodal distributions: YRI07 (friends have skipped school) and YRI09 (friends bully others, which was also zero-inflated). When possible, item recoding was avoided to retain maximum item information and retain consistency with prior research and uses, and because the YRI has traditionally been scored using item means (32, 33). Items with extreme L-shape distributions were recoded for consistency with factor model assumptions (56, 67) while balancing the retention of the variance in responses against severely violating normality assumptions as much as possible (i.e., maintaining skewness at less than ±2 and kurtosis between −4 and 10). Specifically, YRI02 was trichotomized while YRI10, YRI12, YRI21, and YRI23 were dichotomized. In addition, a log link function modeled count items (YRI6–YRI11), except YRI10, which had been dichotomized.

#### 2.3.2 Latent structure: factor analysis

Figure 1 presents the two latent structure models that were compared using relative indices of model fit based on Akaike's Information Criterion (AIC), n-adjusted Bayesian Information Criterion (nBIC), log-likelihood (LL), and inspection of factor loadings. Confirmatory factor analyses were conducted using maximum likelihood with robust standard errors (MLR), assuming normal distributions for all factors, which unfortunately does not provide indices of absolute model fit. The alternative weighted least squares with mean and variance adjustments estimator is not appropriate for count data. The best-fitting model factor loadings were estimated for the aggregate sample, then separately by sex (boys vs. girls) and again separately by YRI application purpose (research vs. screening for intervention) to investigate any latent structure differences.

#### 2.3.3 Latent structure: measurement invariance

After determining the YRI's best-fitting latent structure, measurement invariance was tested first between sexes and then between YRI application purposes using moderated non-linear factor analyses (68, 69). Measurement invariance testing was conducted by sequentially comparing the fit to the metric invariance, configural invariance, and scalar invariance data. Competing models were compared using the Rutkowski and Svetina (70) criteria for invariance [−0.02 comparative fit index (CFI) change and 0.03 root mean square error of approximation (RMSEA) change for metric; −0.01 CFI and 0.01 RMSEA for scalar].

When non-invariance was found, we identified the item(s) that contributed to the non-invariance. For each model, we constrained the variance of the factor to one and freely estimated all the factor loadings. When testing for partial metric invariance, we constrained parameters for all but one item at a time. After we identified partial metric invariance, we tested scalar invariance only for the items for which metric invariance was satisfied. Testing for measurement non-invariance involves many hypothesis-free statistical tests, so we used a criterion of *α* = 0.01 to reject the null hypothesis of invariance to balance Type I and Type II error rates.

#### 2.3.4 Validity: concurrent and predictive analyses

The YRI total and subscale scores’ concurrent and predictive validity were estimated using bivariate associations, with the specific statistic based on the distribution of the validity criterion (Pearson’s *r*, Spearman’s rho, or odds ratio). To estimate YRI thresholds’ improvement over chance as a screening tool, the area under the receiver operating characteristic curve was estimated (71). To preliminarily evaluate the impact of revising the traditional YRI scoring algorithms to account for non-invariance, associations were estimated for both the traditional scores and adjusted scores accounting for non-invariance. Validity analyses were conducted separately within study samples because the specific validity criteria varied among samples, to replicate validity tests, and to inspect the robustness of bivariate associations across different populations. Analyses of neurocognitive functioning were considered preliminary due to the relatively small number of participants, emphasizing effect sizes rather than *p*-values, and were repeated using multiple regression to statistically control for age as a proxy for developmental variability among participants. Single analysis and/or Benjamini and Hochberg’s false discovery rate (72) *p*-values were presented.

## 3 Results

### 3.1 Participants

Overall, the aggregate sample was diverse in sex, race, and ethnicity. The four samples analyzed for YRI latent structure were each composed of approximately half girls and boys. As noted, demographics other than sex were not collected from the Sixth Graders Sample, but screening occurred in diverse school districts, and youths were approximately 12 years old on average. The Well-child Check-up sample had a greater proportion of Black youths than other samples (*p* < 0.001), whereas the Chronic Stress sample had a greater proportion of White youths (*p* < 0.001). Compared to the Standardization and Sixth Graders samples, the Chronic Stress and Well-child Check-up samples had greater mean YRI scores (all Dunnett T3 multiple comparisons test, *p* < 0.001).

#### 3.2.1 Latent structure: factor analysis

In the aggregated sample, the Liability and Subscales model better fit the data (AIC = 120,353; nBIC = 120,529; LL = −60,112.49; *df* = 64) than the Overall Liability model (AIC = 123,190; nBIC = 123,551; LL = −61,533.77; *df* = 61); the two models were not nested, precluding a likelihood ratio *χ*^2^ comparison. The Liability and Subscales standardized factor loadings are shown in Table 3. Each first-order factor loaded at least 0.7 onto Liability in the aggregated sample. At the item level, the Disinhibition item loadings were greater than 0.4. The Peer Conduct Problems item loadings were larger at 0.6 or greater. The Social Contagion item loadings were at least 0.5 except for YRI20 (loading = 0.343). When estimated separately for boys vs. girls or research vs. screening purposes using unconstrained two-group models, all factor loadings closely resembled results from the aggregated sample, suggesting that the Liability and Subscales model offers a robust latent structure (configural invariance) across subgroups.

**Table 3:** *Youth Risk Index* standardized factor loadings for the best-fitting factor model for the aggregated sample by sex and purpose, respectively.

Factor	Item	Aggregated sample	Sex		Purpose	
			Boys	Girls	Research	Screening
Disinhibition		0.82	0.87	0.78	0.76	0.87
	YRI01	0.57	0.60	0.54	0.59	0.52
	YRI02^a^	0.57	0.60	0.56	0.66	0.44
	YRI03	0.57	0.56	0.59	0.66	0.44
	YRI04	0.58	0.60	0.55	0.64	0.50
	YRI05	0.42	0.39	0.45	0.44	0.36
	YRI13	0.54	0.57	0.52	0.56	0.58
	YRI14	0.60	0.63	0.58	0.62	0.63
	YRI15	0.57	0.58	0.57	0.55	0.57
	YRI16	0.60	0.62	0.57	0.58	0.65
	YRI17	0.47	0.48	0.46	0.34	0.60
	YRI18	0.54	0.55	0.53	0.46	0.59
	YRI19	0.67	0.69	0.65	0.64	0.68
Peer conduct problems		0.71	0.65	0.74	0.71	0.69
	YRI06^a^	0.98	1.08	0.87	0.93	0.96
	YRI07^a^	1.44	1.43	1.45	1.52	1.35
	YRI08^a^	0.96	1.05	0.86	0.88	0.96
	YRI09^a^	1.51	1.54	1.50	1.30	1.65
	YRI10^a^	0.81	0.84	0.79	0.79	0.81
	YRI11^a^	1.85	2.05	1.68	1.66	1.90
Social contagion		0.83	0.82	0.83	0.85	0.84
	YRI12^a^	0.59	0.59	0.58	0.59	0.58
	YRI20	0.35	0.40	0.31	0.37	0.35
	YRI21^a^	0.77	0.73	0.80	0.75	0.79
	YRI22	0.59	0.63	0.56	0.57	0.62
	YRI23^a^	0.86	0.86	0.86	0.85	0.85

“Purpose” indicates why the youths completed the YRI (for research or screening). All factor loadings reached *p* <. 001. Loadings corresponding to disinhibition, peer conduct disorder behaviors, and social contagion represent the loadings of these lower-order factors onto the higher-order risk construct.

^a^ Standardized subfactor loadings are in logit, cumulative logit, or log scale.

#### 3.2.2 Latent structure: measurement invariance

Measurement invariance was tested separately for each latent factor because screening traditionally is based on YRI's total score (i.e., Overall Liability), while interpreting each first-order subscale enhances the information garnered from a youth's screening results. In addition, the fit of the second-order factor is identical to the fit of the three-factor model with correlated factors (because the three off-diagonal elements of the factor correlation matrix are reparametrized into the three second-order loadings). As such, the second-order factor also supports the three-first-order-factor model as a better fit to the data than the traditional single first-order factor. The primary benefit of the second-order factor is that it allows estimation of the higher-order factor score, which incorporates differential loadings at the item and first-order factor levels. Moreover, the second-order factor corresponds to the traditional overall YRI score on which screening decisions are based. Since the data do not support item-level unidimensionality, we explored invariance for each of the three first-order factors in addition to the second-order factor.

For Overall Liability, measurement invariance was found between sexes, but partial scalar non-invariance occurred between purposes (Table 4). Compared to applying the YRI for research, when used for screening, the YRI scores were lower by 0.367 on average. Differential item functioning occurred for two items, YRI13 and YRI14, both of which were originally designed to measure distractibility within school settings. When the YRI13 and YRI14 intercepts were free to vary, measurement invariance was met for the remaining YRI items (the change in fit from metric to scalar invariance was −0.017 for CFI and 0.000 for RMSEA).

**Table 4:** Measurement invariance of the *Youth Risk Index's* overall liability.

*Model*: Fit index	Invariance type			
	Configural	Metric	Scalar	Partial scalar^a^
Sex				
*χ* ^2^	6,093.586	6,239.410	6,608.303	—
*df*	460	483	505	—
CFI	0.747	0.742	0.726	—
RMSEA	0.075	0.074	0.075	—
SRMR	0.070	0.074	0.074	—
Δ *df*	—	23	22	—
Δ *χ*^2^	—	145.824^b^	368.893^b^	—
Δ CFI	—	−0.005	−0.016	—
Δ RMSEA	—	−0.001	0.001	—
Δ SRMR	—	0.004	0.000	—
Purpose				
*χ*2	5,732.261	6,252.640	7,005.655	6,646.414
*df*	460	483	505	503
CFI	0.760	0.737	0.704	0.72
RMSEA	0.072	0.074	0.076	0.074
SRMR	0.071	0.090	0.092	0.092
Δ *df*	—	23	22	20
Δ *χ*^2^	—	520.379^b^	753.015^b^	393.774^b^
Δ CFI	—	−0.023	−0.033^c^	−0.017
Δ RMSEA	—	0.002	0.002	0.000
Δ SRMR	—	0.019	0.002	0.002

Configural invariance indicates that the general latent structure (number of factors and significant item loadings) does not differ across groups. Metric invariance indicates equivalent factor loadings across groups. Scalar invariance indicates equivalent factor loadings and intercepts across groups. Partial invariance indicates that specific items are identified as the source(s) of metric or scalar non-invariance so that subsequent adaptations to the measure can account for the non-invariance.

SMSR, Square root mean square residual.

^a^ Rutkoski and Svetina’s (70) criterion was met after freeing YRI13 and YRI14 to vary between research and screening purposes.

^b^ *p* < 0.001 (metric: −0.023 CFI change and 0.002 RMSEA change; scalar: −0.017 CFI change and 0.000 RMSEA change).

^c^ Failed to meet a Rutkoski and Svetina (70) criterion for invariance (−0.02 CFI change and 0.03 RMSEA change for metric; −0.01 CFI and 0.01 RMSEA for scalar);

When evaluating first-order factor scores in isolation, more items contributed to partial non-invariance compared to Overall Liability (Tables 5, 6). For Disinhibition, YRI01 had partial metric non-invariance between sexes and purposes. The three items for Peer Conduct Problems had partial metric and scalar non-invariance across purposes. Social Contagion's YRI12 had metric invariance across sexes and purposes, while YRI20 and YRI23 had metric invariance across sexes. The current results cannot indicate whether scoring refinements to account for non-invariance due to these items might meaningfully and clinically improve YRI scoring because even non-consequential differential item functioning may be statistically significant with a large enough sample size (74, 75).

**Table 5:** Measurement invariance of the *Youth Risk Index's* first-order factors.

Fit statistic	Invariance by sex			Invariance by purpose		
	Configural	Metric	Scalar	Configural	Metric	Scalar
Disinhibition						
LL	−58,799.30	−59,105.72	−59,256.02	−59,108.87	−59,777.87	−60,062.27
Parameters	75	63	50	75	63	50
AIC	1,17,748.59	1,18,337.44	1,18,612.03	1,18,367.74	1,19,681.73	1,20,224.54
nBIC	1,17,987.84	1,18,538.41	1,18,771.53	1,18,608.08	1,19,883.61	1,20,384.76
*p*-value	—	<0.001	<0.001	—	<0.001	<0.001
Peer conduct problems						
LL	−22,373.92	−22,383.79	−22,390.03	−22,653.36	−22,665.93	−22,696.44
Parameters	23	18	13	23	18	13
AIC	44,793.83	44,803.57	44,806.07	45,352.72	45,367.86	45,418.88
nBIC	44,866.24	44,860.23	44,846.99	45,425.45	45,424.78	45,460.00
*p*-value	—	0.00	0.07	—	<0.001	<0.001
Social contagion						
LL	−14,795.56	−14,802.30	−14,806.84	−14,952.68	−14,957.55	−14,986.32
Parameters	27	22	17	27	22	17
AIC	29,645.11	29,648.61	29,647.68	29,959.37	29,959.11	30,006.63
nBIC	29,731.32	29,718.85	29,701.96	30,045.97	30,029.67	30,061.16
*p*-value	—	0.01	0.05	—	0.04	<0.001

The *p*-values are for the Satorra–Bentler-corrected likelihood ratio tests comparing more constrained models to the adjacent less-constrained models (73).

**Table 6:** Items yielding the measurement invariance of the *Youth Risk Index's* first-order factors.

Item	Sex		Purpose	
	Loading	Intercept/threshold(s)	Loading	Intercept/threshold(s)
Disinhibition				
YRI01	Girls > boys^a^	Girls > boys	Research > screening^a^	Screening > research
YRI02				
YRI03				
YRI04				
YRI05				
YRI13				
YRI14				
YRI15				
YRI16				
YRI17				
YRI18				
YRI19				
Peer conduct problems				
YRI06		—		
YRI07		—		
YRI08		—		
YRI09		—	Screening > research^a^	Research > screening
YRI10		—		Screening > research^a^
YRI11		—		Research > screening^a^
Social contagion				
YRI12	Girls > boys^a^	Girls > boys	Research > screening^a^	Screening > research
YRI20	Girls > boys^a^	Boys > girls		
YRI21		—		
YRI22		—		
YRI23	Boys > girls^a^	Girls > boys		

— indicates that partial threshold invariance was not tested for these items because the fully scalar invariant model did not fit significantly worse than the fully metric invariant model.

^a^ Non-invariant effects are significant after applying the Benjamini–Hochberg correction (72).

Although full practical impact analyses were beyond the scope of this study (75, 76), a preliminary evaluation of the impact of differential item functioning was conducted by comparing traditional YRI scores to scores that were adjusted to account for non-invariance, which were both standardized for comparisons in the same metric. Means and ranges were similar for traditional and adjusted scores, respectively ($\bar{X}$ = 0.00 for both; minimum = −1.41 vs. −1.46; maximum = 3.29 vs. 4.93). The differences between individuals’ traditional and adjusted scores were negligible on average ($\bar{X}$ = 0.04, SD = 0.28) but were sizable for some youths (ranging from −0.77 to 2.07). Similar results were found for the three first-order factors (Table 7). Overall, the score distributions differed little between traditional and adjusted scoring, whereas the adjusted score distributions had smaller ranges, with the greatest differences in persons who had high liability scores using the traditional YRI algorithm.

**Table 7:** Comparison of the distributions of traditional *Youth Risk Index* standardized scores vs. standardized scores that are adjusted for DIF.

Descriptive statistic	Overall liability	Disinhibition	Peer conduct problems	Social contagion
Descriptive statistics of traditional vs. adjusted score distributions				
$\bar{X}$	0.00 vs. −0.02	0.00 vs. 0.04	0.00 vs. −0.15	0.00 vs. −0.01
*SD*	1.00 vs. 0.86	1.00 vs. 0.92	1.00 vs. 0.88	1.00 vs. 0.74
Minimum	−1.46 vs. −1.41	−1.57 vs. −1.44	−0.81 vs. −1.47	−0.80 vs. −0.75
Maximum	4.93 vs. 3.29	4.09 vs. 3.52	4.00 vs. 1.64	5.56 vs. 2.74
Differences between individuals' scores				
$\bar{X}$	0.04	−0.04	0.17	0.01
*SD*	0.28	0.18	0.47	0.41
Minimum	−0.77	−0.46	−0.81	−1.13
Maximum	2.07	0.73	2.41	2.97

The traditional and DIF-adjusted YRI scores were first converted to standard scores for comparisons in the same metric. *N* = 3,375 because participants with more than four missing items were excluded. Difference scores were computed using Traditional_Standard Score_ – Adjusted_Standard Score_.

The rank order of scores changed little due to adjusting for non-invariance, as the two scoring algorithms correlated highly for Overall Liability (Pearson’s *r* = 0.96), Disinhibition (*r* = 0.99), Peer Conduct Problems (*r* = 0.89), and Social Contagion (*r* = 0.93). Similarly, large correlations were found within both sexes and purposes (Table 8). Hence, predictive accuracy and validity were reported when only using traditional YRI scores.

**Table 8:** Correlations between traditional scores and scores adjusted for non-invariance.

Participants	Overall liability	Disinhibition	Peer conduct problems	Social contagion
*r* between adjusted and non-adjusted scores				
Aggregate sample	0.96	0.99	0.89	0.93
Girls	0.97	0.99	0.89	0.94
Boys	0.96	0.98	0.88	0.93
Research purpose	0.97	0.99	0.89	0.93
Screening purpose	0.96	0.99	0.91	0.94

Prior to comparing the traditional and non-invariance-adjusted scores, they were both standardized with mean = 0 and SD = 1. *r* = Pearson’s correlation, all of which reached *p* < 0.001.

#### 3.3.1 Validity: criterion validity

YRI total score (when used for screening) associations were large for SU and conduct problems (2+ conduct disorder behaviors), both concurrently and up to 1 year later (Table 9). Overall predictive accuracy was good in all analyses, based on the area under the receiver operating characteristic curves, with stronger accuracy for conduct problems compared to SU. The results observed in the Chronic Stress sample, in which the YRI items were originally identified, were replicated in the general population (Standardization) and clinical (Well-child Check-up) samples. Moreover, similar results were observed between youths who identified as girls vs. boys (Table 9).

**Table 9:** Concurrent and predictive criterion validity and area under the receiver operating characteristic curve of the *Youth Risk Index* in three sample**s.**

Participants	Substance use				Conduct problems			
	Concurrent		By 1 year later		Concurrent		By 1 year later	
	OR (CI)	ROC (SE)	OR (CI)	ROC (SE)	OR (CI)	ROC (SE)	OR (CI)	ROC (SE)
ALEXSA-R standardization								
Aggregate	5.0 (3.8–6.6)	0.73 (0.02)	—	—	44.7 (23.8–84.0)	0.88 (0.01)	—	—
Girls (*n* = 584)	6.9 (4.6–10.4)	0.78 (0.02)	—	—	45.7 (17.9–117.0)	0.89 (0.02)	—	—
Boys (*n* = 569)	4.1 (2.8–5.9)	0.70 (0.02)	—	—	39.8 (16.8–94.2)	0.87 (0.02)	—	—
Chronic Stress								
Aggregate	6.7 (3.7–12.1)	0.78 (0.03)	3.8 (2.3–6.1)	0.71 (0.03)	18.3 (9.2–36.3)	0.85 (0.03)	7.6 (4.1–14.2)	0.75 (0.03)
Girls (*n* = 308)	8.7 (3.4–22.4)	0.76 (0.06)	5.8 (2.6–12.5)	0.72 (0.04)	69.1 (15.8–302.6)	0.90 (0.03)	17.2 (5.3–55.9)	0.81 (0.04)
Boys (*n* = 331)	5.1 (2.4–11.1)	0.74 (0.04)	2.6 (1.4–4.8)	0.65 (0.04)	8.9 (4.1–20.0)	0.78 (0.04)	4.0 (1.9–8.6)	0.68 (0.04)
Well-child Check-up								
Aggregate	3.1 (2.0–4.9)	0.67 (0.03)	2.7 (1.7–4.1)	0.64 (0.03)	7.6 (4.5–13.1)	0.78 (0.03)	5.1 (3.1–8.3)	0.71 (0.03)
Girls (*n* = 186)	3.8 (2.0–7.2)	0.69 (0.04)	3.2 (1.7–6.0)	0.66 (0.04)	7.8 (3.6–16.7)	0.77 (0.04)	5.0 (2.5–10.0)	0.72 (0.04)
Boys (*n* = 171)	2.6 (1.4–4.8)	0.65 (0.05)	2.1 (1.2–3.9)	0.62 (0.04)	7.4 (3.5–15.9)	0.78 (0.04)	5.1 (2.5–10.3)	0.71 (0.04)

OR, odds ratio; CI, confidence interval; ROC, area under the receiver operating characteristic curve; SE, standard error.

Substance use = any lifetime use of alcohol, tobacco, or cannabis. Conduct problems = lifetime experiencing of two or more conduct disorder behaviors. YRI items were originally derived from the Chronic Stress sample based on how strongly an item predicted SU and/or conduct problems 1 year later.

#### 3.3.2 Validity: convergent and discriminant analyses

Table 10 presents bivariate associations between YRI scores and youth self-reports of well-known risks and resiliencies to SU and conduct problems. YRI scores correlated positively with risk factors (Sensation Seeking, Parental Problems with the Law, Parental Problems from SU, and Depression symptoms), with mostly medium-sized associations. YRI scores correlated negatively with resiliencies (Self-management, Parent Fortification, and School Protection), with small to medium-sized coefficients. In addition to predicting binary coding of any SU and conduct problems (2+ conduct disorder behaviors) (Table 9), the YRI scores correlated with the Counts of the Number of Different Substances Used and Conduct Disorder Behaviors. No consistent differential associations with vulnerabilities or resiliencies were observed across the YRI first-order factor scores.

**Table 10:** Concurrent validity of unadjusted *Youth Risk Index* scores with risk factors for early adolescent substance use.

Validity comparator	Overall liability	Disinhibition	Peer conduct problems	Social contagion
ALEXSA-R Standardization sample				
ALEXSA-R measures (youth reports)				
Self-management domain (*α* = 0.75)	−0.33***	−0.35***	−0.14**	−0.28***
Sensation seeking domain (*α* = 0.82)	0.52***	0.38***	0.42***	0.61***
Parent fortification domain (*α* = 0.89)	−0.51***	−0.46***	−0.37***	−0.39***
Parental problems with the law^a^	0.30^b^^,^***	0.29^b^^,^***	0.23**	0.14^b^^,^**
Parental problems from substance use^b^	0.17^b^^,^**	0.14^b^^,^**	0.17^b^^,^**	0.10^b^^,^**
Depression (*α* = 0.87)	0.49***	0.48***	0.39***	0.20**
Count of substances used (alcohol, tobacco, cannabis, inhalants)	0.27^b^^,^***	0.21^b^^,^**	0.15^b^^,^**	0.38^b^^,^***
Count of conduct disorder behaviors	0.63^b^^,^***	0.56^b^^,^***	0.49^b^^,^***	0.44^b^^,^***
Chronic stress sample				
ALEXSA-R measures (youth reports)				
Self-management domain (*α* = 0.78)	−0.44***	−0.45***	−0.20**	−0.29***
Sensation seeking domain (*α* = 0.73)	0.33***	0.21**	0.25***	0.38***
School protection domain (*α* = 0.66)	−0.29***	−0.25***	−0.17**	−0.26***
Parent fortification domain (*α* = 0.86)	−0.32***	−0.25***	−0.27***	−0.20**
Parental problems with the law^a^	0.31^b^^,^***	0.27^b^^,^***	0.24^b^^,^***	0.23^b^^,^***
Parental problems from substance use^a^	0.28^b^^,^***	0.23^b^^,^***	0.23^b^^,^***	0.23^b^^,^***
Depression (*α* = 0.87)	0.41***	0.42***	0.28***	0.04
Count of substances used (alcohol, tobacco)	0.45^b^^,^***	0.35^b^^,^***	0.35^b^^,^***	0.37^b^^,^***
Count of conduct disorder behaviors	0.66^b^^,^***	0.42^b^^,^***	0.37^b^^,^***	0.55^b^^,^***
Well-child Check-up sample				
ALEXSA-R measures (Youth reports)				
Self-management domain (*α* = 0.69)	−0.25***	−0.29***	−0.08	−0.29***
Sensation seeking domain (*α* = 0.79)	0.38***	0.27***	0.32***	0.38***
Parent fortification domain (*α* = 0.89)	−0.32***	−0.29***	−0.16	−0.40***
Depression (*α* = 0.88)	0.35***	0.38***	0.21	0.20**
Count of substances used (alcohol, tobacco, cannabis)	0.31^b^^,^***	0.23^b^^,^**	0.27^b^^,^***	0.29^b^^,^***
Count of conduct disorder behaviors	0.69^b^^,^***	0.51^b^^,^***	0.67^b^^,^***	0.49^b^^,^***
Parent ratings				
CBCL: aggression	0.11*	0.19**	0.02	0.05
Attention problems	0.10	0.13*	0.06	0.01
Conduct	0.15*	0.23**	0.03	0.07
Rule breaking	0.16*	0.25***	0.04	0.06
Withdrawn/depressed	0.07	0.10	0.01	0.01
Anxious	0.04	0.09	0.04	−0.06
Parental monitoring (*α* = 0.80)	−0.16*	−0.14*	−0.09	−0.07
Parent supports good behavior (*α* = 0.76)	−0.02	−0.06	0.03	0.00
Parental rule setting (*α* = 0.78)	−0.10	−0.07	−0.07	−0.02

*α*, Cronbach's alpha internal consistency (measures lacking an *α* either did not have item-level data available, were single-item measures, or didn't meet distributional assumptions); CBCL, ASEBA *Child Behavior Checklist*, parent rating scales (60).

^a^ Binary variable.

^b^ *ρ*, Spearman's rho.

Cell entries are Pearson’s correlations unless otherwise noted.

* *p* < 0.05; **p < 0.01; ***p < 0.001.

As expected, compared to youth self-reports, the YRI scores correlated to a smaller degree with parent reports of youths’ externalizing in the Well-child Check-up sample (Table 10). Parent reports of youth externalizing correlated strongest with Disinhibition subscale scores, even compared to Overall Liability. For the parent reports of their own parenting, only Parental Monitoring correlated negatively with YRI Overall and Disinhibition scores (small effect sizes). Unlike the youth reports of Depression, the YRI scores did not correlate with parent reports of youths’ internalizing symptoms.

#### 3.3.3 Validity: neurocognitive functioning

In the CogNIT sample, the YRI Overall Liability scores were associated with several measures of cognitive functioning with small to moderate effect sizes (albeit needing a larger sample size to attain traditional critical *p*-values) (Table 11). After controlling for age as a proxy measure of the considerable developmental maturation in both cognitive functioning and liability to SU/SUD that occurs from ages 9 to 17, the Overall Liability scores were still associated with performance on the Planning/Sequencing Tower and the 0-back fractal test with medium-sized associations (correcting for multiple analyses using the false discovery rate). Importantly, controlling for age presents a stringent criterion because age encompasses not only maturation in cognition but also many increasing age-related risk factors that are known to be strongly associated with SU/SUD (35).

**Table 11:** The *Youth Risk Index’s* concurrent validity with neurocognitive measures of attention, executive functioning, and working memory.

Variables	*n*	Mean	SD	Bivariate *r*	Multiple regression		
					Multiple *R*_age, YRI_	Partial *r*_YRI_	Partial *r*_age_
WASI digit span forward	25	10.44	2.97	0.38	0.55^a^	0.13	0.47
D-KEFS							
Color-word inhibition/switching	35	9.86	2.98	0.21	0.46	0.05	0.44
Planning/sequencing tower	35	16.31	3.23	0.52^b^	0.58^b^	0.40	0.27
Fractal *N*-back test							
0-back, pre-stressor	27	0.78	0.20	0.35	0.65^b^	−0.56	0.58
2-back, pre-stressor	27	0.44	0.22	0.23	0.38	0.03	0.37
Emotional *N*-back test *d*′	27	2.06	0.68	0.18	0.39	−0.08	0.43

Multiple regression analyses included age and YRI scores as predictors. Bivariate *r* results are regressions of YRI scores on the row variable. Partial *r* results present partial coefficients (standardized *b*) from the multiple regression of a row variable on predictors of age and YRI scores.

^a^ False discovery rate-corrected *p* < .05 (72).

^b^ False discovery rate-corrected *p* < .01.

## 4 Discussion

To summarize, we evaluated the YRI's latent structure, measurement invariance, and several forms of validity using data from multiple samples of youth who were recruited for different study purposes. The results consistently documented strong psychometric properties for Overall Liability and the three first-order factor scores. The results identified certain items to have statistical non-invariance between sexes and/or testing purposes, which yielded little impact on the rank ordering of the scores (and thus the validity of the YRI factor scores). Even so, some individuals’ standard scores changed considerably between the traditional and non-invariance-adjusted scores. In the future, complete impact analyses could determine whether and what corrections may be needed for scoring YRI factor scores based on non-invariance results (74–76). Until impact analyses are completed, caution is warranted regarding the interpretation of first-order YRI factor scores. While the Overall Liability scores had the best measurement properties, supporting the traditional screening approach using information from all the YRI items, accounting for non-invariance in YRI13 and YRI14 might improve the generalization of research findings when using the total YRI score for screening purposes. All the YRI factor scores consistently demonstrated concurrent and predictive validity for SU, conduct problems, risk and resiliency factors, depression, working memory, and attention. The YRI's predictive accuracy was better for conduct problems than for SU, likely due in part to varying accessibility to substances among youths and the lower prevalence of SU than conduct problems in the age groups of our samples.

### 4.1 Non-invariance by sex

Understanding and accounting for the non-invariance observed between girls and boys for Disinhibition and Social Contagion may facilitate linking sex-specific screening and prevention strategies with epidemiology trends. Historically, pronounced sex differences have been observed in adolescent and young adult SU/SUD prevalence and etiology in the US; however, these differences have diminished over the last two decades (77–79). Less evidence is available regarding sex differences in SU/SUD overall liability during late childhood and early adolescence. Our results suggest that in cohorts from the last 10 years, little difference exists between youths who identify as boys or girls in their average overall liability and in the risk stemming specifically from disinhibition and peer conduct problems, whereas greater differences exist between them regarding social contagion. Delineating sex differences in liability to SU/SUD during late childhood and early adolescence has the potential to reveal important insights for screening and prevention strategies (79, 80). If the sex differences are meaningful and can be delineated within YRI screening, forecasting accuracy may be improved and risk factor profiling to individualize prevention strategies could be adopted accordingly.

### 4.2 SU/SUD liability

First and foremost, YRI scores are highly associated with initiating SU before high school (whether prior to or after the YRI is completed). In addition to documenting the concurrent and predictive validity of the YRI, our convergent validity results demonstrate the scope of liability to SU/SUD that the YRI scores epitomize. The breadth of SU/SUD risk and resilience factors that YRI scores account for include behavior problems, disinhibition, depression, sensation seeking, familial substance use problems, parental problems with the law, friends’ behavior problems, protective parenting practices, protective self-management behaviors, neurocognitive risk, and vulnerability to social contagion. Encapsulating this breadth of liability sources is essential to accurately measure overall liability prior to SU/SUD-related problems or even SU initiation because of the highly diverse trajectories leading to the equifinality of SUD.

### 4.3 Neurocognitive risk

The correlation between YRI Overall Scores and D-KEFS Tower total performance suggests that YRI scores discriminate among levels in planning and problem-solving that are associated with SU/SUD liability. Remarkably, despite the small sample, this correlation maintained a *p-*value < 0.05 even with age as a covariate. Regression analysis with the 0-back pre-stress task performance suggested that the low-load working memory deficits that are associated with SU/SUD risk are reflected in YRI scores, although this association did not maintain *p* < 0.05 after accounting for maturational changes from ages 9 to 17. The specific characteristics that the YRI items query and are associated with include deficits in planning, problem-solving, working memory, impulsivity, anger coping, distractibility, and friends' behavior problems (54, 81–83). Future research that clarifies which ALEXSA subscale scores are associated with specific underlying neurophysiological and genetic risks could facilitate identification and primary prevention to counter individuals’ biological predispositions toward SU/SUD. To illustrate, certain ALEXSA profiles of risks may reveal neurobiological risk without requiring neuroimaging or genetic testing.

An ongoing longitudinal study led by this research team is investigating youths, aged 12–14, who are experiencing anxiety to identify factors that are associated with trajectories toward and away from SU through middle adolescence (R01DA057312, Fishbein, Belger, and Ridenour, MPIs). Stratified recruitment is in part based on the youths’ YRI scores. Maturation of functional brain connectivity, emphasizing fronto-limbic subsystems and physiological stress responsivity, and well-known risk factors will be studied over 24-month follow-ups. We anticipate that data from this study will clarify associations between YRI scores and underlying neurobiological risk for SU/SUD.

### 4.4 Limitations

The multiple strengths of this study include large sample sizes representing epidemiological, clinical, and enriched at-risk youths; balanced subsample proportions of sexes and YRI purposes to evaluate measurement invariance; and varied criteria for testing validity. Even so, the results ought to be interpreted within the context of the study’s limitations to help guide future research. While this study was the first to investigate subscales for the YRI, the results indicated that further evaluation of the partial non-invariance is needed before the subscale scores can be interpreted for clinical purposes. Specifically, impact analyses are needed to evaluate if there is a need for, and how to, resolve non-invariance associated with sex and YRI purpose. In addition, because of the small CogNIT sample size, the YRI subscale scores were not reported, but we expect they will be differentially associated with specific neurocognitive deficits and related neurological underpinnings. Finally, YRI scores’ predictive accuracy was only assessed for 1-year outcomes; longer-term follow-ups in age groups when SU becomes more prevalent could improve screening thresholds to identify which youths may benefit from primary prevention.

### 4.5 Next steps

In addition to addressing this study's limitations, our results support pursuing several lines of investigation to elevate the YRI's utility as a screening tool for selective/indicated primary prevention. Fully developing each of the first-order factors/subscales could aid in matching a prevention strategy to individual youths’ sources of risk, although a broader assessment of risk factors (e.g., the ALEXSA-R) following screening is more likely to succeed in coupling youths’ individual needs to an impactful program (79, 84, 85). To illustrate, a similar approach of matching intervention programs to personality type in middle adolescents has effectiveness in reducing rates of harmful alcohol use (86). Numerous efficacious prevention programs exist for pre- or early adolescents that target specific sources of risk, such as emotional and behavioral disinhibition (45, 46, 87), reactive aggression and anger coping (88, 89), parenting skills for at-risk youths (33, 90), peer network influences (91), and boredom (92, 93). However, there is a dearth of studies that have evaluated the efficacy of offering multiple prevention programs or of matching programs to youths based on their specific sources of risk for SU/SUD. The ongoing research by our team will investigate the associations among YRI scores, ALEXSA subscales, and variations in fronto-limbic functioning and physiological stress responsivity. Finally, based on the broad mediating role(s) that SU/SUD plays in exacerbating many medical and mental illnesses (5–8), it is important to evaluate the potential role(s) that YRI screening could play in the prevention of or early intervention for mental illness.

## Data Availability

The datasets presented in this article are not readily available because the data may be available by request from the first author. Requests to access the datasets should be directed to Ty Ridenour, RidenourTy@gmail.com.

## References

[1] Cook JL. The opioid epidemic. Best Pract Res Clin Obstet Gynaecol. (2022) 85:53–8. 10.1016/j.bpobgyn.2022.07.003 36045027

[2] Farrell M Martin NK Stockings E Bórquez A Cepeda JA Degenhardt L Responding to global stimulant use: challenges and opportunities. Lancet. (2019) 394:1652–67. 10.1016/S0140-6736(19)32230-5 31668409 PMC6924572

[3] Pacurucu-Castillo SF Ordóñez-Mancheno JM Hernández-Cruz A Alarcón RD. World opioid and substance use epidemic: a Latin American perspective. Psychiatr Res Clin Pract. (2019) 1:32–8. 10.1176/appi.prcp.20180009 36101564 PMC9175731

[4] Ramadan M Ghulam E Alhusseini N. Does illicit amphetamine seizures quantity associated with amphetamine use disorder related admissions in Saudi Arabia? BMC Psychiatry. (2023) 23:23. 10.1186/s12888-023-04523-3 36627601 PMC9830699

[5] Bahorik AL Newhill CE Eack SM. Characterizing the longitudinal patterns of substance use among individuals diagnosed with serious mental illness after psychiatric hospitalization. Addiction. (2013) 108:1259–69. 10.1111/add.12153 23432626 PMC3679358

[6] Homman LE Edwards AC Cho SB Dick DM Kendler KS. Gender and direction of effect of alcohol problems and internalizing symptoms in a longitudinal sample of college students. Subst Use Misuse. (2017) 52:429–38. 10.1080/10826084.2016.1233983 27849409 PMC5601307

[7] Lev-Ran S Roerecke M Le Foll B George TP McKenzie K Rehm J. The association between cannabis use and depression: a systematic review and meta-analysis of longitudinal studies. Psychol Med. (2014) 44:797–810. 10.1017/S0033291713001438 23795762

[8] Moore TH Zammit S Lingford-Hughes A Barnes TR Jones PB Burke M Cannabis use and risk of psychotic or affective mental health outcomes: a systematic review. Lancet. (2007) 370:319–28. 10.1016/S0140-6736(07)61162-3 17662880

[9] Hoffman KA Ponce Terashima J McCarty D. Opioid use disorder and treatment: challenges and opportunities. BMC Health Serv Res. (2019) 19:1–5. 10.1186/s12913-019-4751-4 31767011 PMC6876068

[10] Mattson CL Tanz LJ Quinn K Kariisa M Patel P Davis NL. Trends and geographic patterns in drug and synthetic opioid overdose deaths—United States, 2013–2019. Morb Mortal Wkly Rep. (2021) 70:202–7. 10.15585/mmwr.mm7006a4 PMC7877587 33571180

[11] Castelpietra G Knudsen AK Agardh EE Armocida B Beghi M Iburg KM The burden of mental disorders, substance use disorders and self-harm among young people in Europe, 1990–2019: findings from the global burden of disease study 2019. Lancet Reg Health–Eur. (2022) 16:1–18. 10.1016/j.lanepe.2022.100341 PMC8980870 35392452

[12] Vashishtha R Pennay A Dietze P Marzan MB Room R Livingston M. Trends in adolescent drinking across 39 high-income countries: exploring the timing and magnitude of decline. Eur J Public Health. (2021) 31:424–31. 10.1093/eurpub/ckaa193 33188681

[13] Yu C Chen J. Global burden of substance use disorders among adolescents during 1990–2021 and a forecast for 2022–2030: an analysis for the global burden of disease 2021. BMC Public Health. (2025) 25:1–16. 10.1186/s12889-025-22107-6 40087641 PMC11909827

[14] Ciccarone D. The rise of illicit fentanyls, stimulants and the fourth wave of the opioid overdose crisis. Curr Opin Psychiatry. (2021) 34:344–50. 10.1097/YCO.0000000000000717 33965972 PMC8154745

[15] O’Donnell J. Trends in and characteristics of drug overdose deaths involving illicitly manufactured fentanyls—United States, 2019–2020. Morb Mortal Wkly Rep. (2021) 70:1740–6. 10.15585/mmwr.mm7050e3 PMC8675656 34914673

[16] Grant BF. Age at smoking onset and its association with alcohol consumption and DSM-IV alcohol abuse and dependence: results from the national longitudinal alcohol epidemiologic survey. J Subst Abuse. (1998) 10:59–73. 10.1016/S0899-3289(99)80141-2 9720007

[17] King KM Chassin L. A prospective study of the effects of age of initiation of alcohol and drug use on young adult substance dependence. J Stud Alcohol Drugs. (2007) 68:256–65. 10.15288/jsad.2007.68.256 17286344

[18] Odgers CL Caspi A Nagin DS Piquero AR Slutske WS Milne BJ Is it important to prevent early exposure to drugs and alcohol among adolescents? Prev Sci. (2008) 19:1037–44. 10.1111/j.1467-9280.2008.02196.x PMC3664402 19000215

[19] Pitkänen T Kokko K Lyyra AL Pulkkinen L. A developmental approach to alcohol drinking behaviour in adulthood: a follow-up study from age 8 to age 42. Addiction. (2008) 103:48–68. 10.1111/j.1360-0443.2008.02176.x 18426540

[20] Rabinowitz JA Jin J Kuo SI Campos AI Renteria ME Huhn AS Positive associations between cannabis and alcohol use polygenic risk scores and phenotypic opioid misuse among African-Americans. PLoS. (2022) 17:e0266384. 10.1371/journal.pone.0266384 PMC8993003 35395044

[21] Thrul J Reboussin BA Rabinowitz JA Maher BS Ialongo NS. Alcohol trajectories and subsequent risk for opioid misuse in a cohort of urban adolescents. Subst Abus. (2021) 42:873–9. 10.1080/08897077.2021.1890675 33759726 PMC8460686

[22] Substance Abuse and Mental Health Services Administration. A Guide to SAMHSA’s Strategic Prevention Framework. Rockville, MD: Center for Substance Abuse Prevention, Substance Abuse and Mental Health Services Administration (2019).

[23] Peiper NC Ridenour TA Hochwalt B Coyne-Beasley T. Overview on prevalence and patterns of substance use in adolescents. Child Adolesc Psychiatr Clin N Am. (2016) 25:349–65. 10.1016/j.chc.2016.03.005 27338960

[24] Ridenour TA Bray BC Scott HS Cottler LB. “Classification and assessment of substance use disorders in adolescents”. In: Essau CA, editor. Adolescent Addiction, Epidemiology, Assessment, and Treatment. New York: Elsevier (2008). p. 17–57.

[25] Centers for Disease Control and Prevention. Youth Risk Behavior Survey Data Summary & Trends Report: 2013–2023. Washington, DC: U.S. Department of Health and Human Services (2024).

[26] Miech RA Johnston LD Patrick ME O’Malley PM Bachman JG Schulenberg JE. Monitoring the Future National Survey Results on Drug use, 1975–2022: Secondary School Students. Monitoring the Future Monograph Series. Ann Arbor, MI: Institute for Social Research, University of Michigan (2023).

[27] Substance Abuse and Mental Health Services Administration. Key Substance Use and Mental Health Indicators in the United States: Results from the 2023 National Survey on Drug Use and Health (HHS Publication No. PEP24-07-021, NSDUH Series H-59). Washington, DC: Center for Behavioral Health Statistics and Quality, Substance Abuse and Mental Health Services Administration (2024).

[28] Conway KP Vullo GC Kennedy AP Finger MS Agrawal A Bjork JM Data compatibility in the addiction sciences: an examination of measure commonality. Drug Alcohol Depend. (2014) 141:153–8. 10.1016/j.drugalcdep.2014.04.029 24954640 PMC4096981

[29] Donovan DM Bigelow GE Brigham GS Carroll KM Cohen AJ Gardin JG Primary outcome indices in illicit drug dependence treatment research: systematic approach to selection and measurement of drug use end-points in clinical trials. Addiction. (2012) 107:694–708. 10.1111/j.1360-0443.2011.03473.x 21781202 PMC3537825

[30] Loflin MJ Kiluk BD Huestis MA Aklin WM Budney AJ Carroll KM The state of clinical outcome assessments for cannabis use disorder clinical trials: a review and research agenda. Drug Alcohol Depend. (2020) 212:107993. 10.1016/j.drugalcdep.2020.107993 32360455 PMC7293929

[31] Stewart RE Cardamone NC Schachter A Becker C McKay JR Becker-Haimes EM. A systematic review of brief, freely accessible, and valid self-report measures for substance use disorders and treatment. Drug Alcohol Depend. (2023) 243:109729. 10.1016/j.drugalcdep.2022.109729 36535096 PMC9872256

[32] Ridenour TA Willis D Bogen DL Novak S Scherer J Reynolds MD Detecting initiation or risk for initiation of substance use before high school during pediatric Well-child Check-ups. Drug Alcohol Depend. (2015) 150:54–62. 10.1016/j.drugalcdep.2015.02.013 25765481 PMC4405881

[33] Galan CA Shaw DS O'Rourke F Reynolds MD Gill A Bogen DL Substance use screening and prevention for adolescents in pediatric primary care: a randomized clinical trial using the family check-up. Res Child Adolesc Psychopathol. (2023) 51:151–63. 10.1007/s10802-022-00978-2 36208361 PMC10146025

[34] Ridenour TA Clark DB Cottler LB. The illustration-based Assessment of Liability and EXposure to Substance use and Antisocial behavior for children. Am J Drug Alcohol Abuse. (2009) 35:242–52. 10.1080/00952990902998715 20180677

[35] Ridenour TA Reid EE Chilenski SM. Developmental momentum and liability to behavioral problems: natural histories of risk factors in youth experiencing chronic stress. Drug Alcohol Depend. (2012) 123:S87–98. 10.1016/j.drugalcdep.2011.12.016 22257754 PMC3342426

[36] Falconer DS. The inheritance of liability to certain diseases, estimated from the incidence among relatives. Ann Hum Genet. (1965) 29:51–76. 10.1111/j.1469-1809.1965.tb00500.x

[37] Vanyukov MM Tarter RE Kirisci L Kirillova GP Maher BS Clark DB. Liability to substance use disorders: 1. Common mechanisms and manifestations. Neurosci Biobehav Rev. (2003) 27:507–15. 10.1016/j.neubiorev.2003.08.002 14599432

[38] Blanco C Rafful C Wall MM Ridenour TA Wang S Kendler KS. Towards a comprehensive developmental model of cannabis use disorders. Addiction. (2014) 109:284–94. 10.1111/add.12382 24261668 PMC3956073

[39] Ridenour TA Minnes S Maldonado-Molina MM Reynolds MD Tarter RE Clark DB. Psychometrics and cross-cultural comparisons of the illustration-based assessment of liability and exposure to substance use and antisocial behavior(c) for children. Open Fam Stud J. (2011b) 4(Suppl 1–M2):17–26. 10.2174/1874922401104010017 22866171 PMC3410723

[40] Leitgöb H Seddig D Asparouhov T Behr D Davidov E De Roover K Measurement invariance in the social sciences: historical development, methodological challenges, state of the art, and future perspectives. Soc Sci Res. (2023) 110:102805. 10.1016/j.ssresearch.2022.102805 36796989

[41] Lees B Garcia AM Debenham J Kirkland AE Bryant BE Mewton L Promising vulnerability markers of substance use and misuse: a review of human neurobehavioral studies. Neuropharmacology. (2021) 187:108500. 10.1016/j.neuropharm.2021.108500 33607147 PMC8129990

[42] Kim-Spoon J Herd T Brieant A Peviani KM Lauharatanahirun N Lee J Bidirectional links between adolescent brain function and substance use moderated by cognitive control. J Child Psychol Psychiatry. (2021) 62:427–36. 10.1111/jcpp.13285 32640083 PMC8124751

[43] Nusslock R Kogan SM Yu T Armstrong CC Chen E Miller GE Higher substance use is associated with low executive control neural activity and higher inflammation. Brain Behav Immun. (2024) 120:532–42. 10.1016/j.bbi.2024.06.018 38925415 PMC12629847

[44] Ivanov I Schulz KP London ED Newcorn JH. Inhibitory control deficits in childhood and risk for substance use disorders: a review. Am J Drug Alcohol Abuse. (2008) 34:239–58. 10.1080/00952990802013334 18428067

[45] Fishbein DH Domitrovich C Williams J Gitukui S Guthrie C Shapiro D Short-term intervention effects of the PATHS curriculum in young low-income children: capitalizing on plasticity. J Prim Prevent. (2016) 37:493–511. 10.1007/s10935-016-0452-5 PMC10505054 27785656

[46] Fishbein DH Novak SP Ridenour TA Thornburg V Hammond J Brown J. Neurocognitive characteristics of early marijuana use initiation in adolescents: a signature mapping analysis. J Stud Alcohol Drugs. (2016) 77:431–40. 10.15288/jsad.2016.77.431 27172575 PMC4869899

[47] Klein RJ Gyorda JA Lekkas D Jacobson NC. Dysregulated emotion and trying substances in childhood: insights from a large nationally representative cohort study. Subst Use Misuse. (2023) 58:1625–33. 10.1080/10826084.2023.2223290 37572018 PMC11000575

[48] Jones SA Del Giacco AC Barnes SJ Nagel BJ. Adolescent substance use is associated with altered brain response during processing of negative emotional stimuli. J Stud Alcohol Drugs. (2023) 84:257–66. 10.15288/jsad.22-00143 36971739 PMC10171254

[49] Wodka EL Loftis C Mostofsky SH Prahme C Larson JC Denckla MB Prediction of ADHD in boys and girls using the D-KEFS. Arch Clin Neuropsychol. (2008) 23:283–93. 10.1016/j.acn.2007.12.004 18243646 PMC2427435

[50] Lindenmuth M Herd T Brieant A Lee J Deater-Deckard K Bickel WK Neural cognitive control moderates the longitudinal link between hedonia and substance use across adolescence. Dev Cogn Neurosci. (2022) 55:101111. 10.1016/j.dcn.2022.101111 35472691 PMC9061620

[51] Fava NM Trucco EM Martz ME Cope LM Jester JM Zucker RA Childhood adversity, externalizing behavior, and substance use in adolescence: mediating effects of anterior cingulate cortex activation during inhibitory errors. Dev Psychopathol. (2019) 31:1439–50. 10.1017/S0954579418001025 30585564 PMC6594917

[52] Quach A Tervo-Clemmens B Foran W Calabro FJ Chung T Clark DB Adolescent development of inhibitory control and substance use vulnerability: a longitudinal neuroimaging study. Dev Cogn Neurosci. (2020) 42:100771. 10.1016/j.dcn.2020.100771 32452466 PMC7038454

[53] Halliburton AE Ridenour TA White BA Deater-Deckard K. Clinically differentiating life-course-persistent and adolescence-limited conduct problems: is age-of-onset really enough? J Appl Dev Psychol. (2017) 52:34–45. 10.1016/j.appdev.2017.06.005 29176919 PMC5699469

[54] Halliburton AE Murray DW Ridenour TA. Interplay among self-regulation processes over time for adolescents in the context of chronic stress. J Cogn Dev. (2024) 25:386–407. 10.1080/15248372.2023.2295894 39149413 PMC11323049

[55] Ridenour TA Caldwell LL Coatsworth DJ Gold MA. Directionality between tolerance of deviance and deviant behavior is age-moderated in chronically stressed youth. J Child Adolesc Subst Abuse. (2011a) 20:184–204. 10.1080/1067828X.2011.555278 PMC3237684 22180721

[56] Curran PJ West SG Finch JF. The robustness of test statistics to nonnormality and specification error in confirmatory factor analysis. Psychol Methods. (1996) 1:16–29. 10.1037/1082-989X.1.1.16

[57] Chilenski SM Ridenour T Bequette AW Caldwell LL. Pathways of influence: how parental behaviors and free time experiences are associated with African American early adolescent development and academic achievement. J Negro Educ. (2015) 84:401–15. 10.7709/jnegroeducation.84.3.0401

[58] Russo MF Stokes GS Lahey BB Christ MAG McBurnett K Loeber R A sensation seeking scale for children: further refinement and psychometric development. J Psychopathol Behav Assess. (1993) 15:69–86. 10.1007/BF00960609

[59] De Los Reyes A. Introduction to the special section: more than measurement error: discovering meaning behind informant discrepancies in clinical assessments of children and adolescents. J Clin Child Adolesc Psychol. (2011) 40:1–9. 10.1080/15374416.2011.533405 21229439

[60] Achenbach TM. Manual for the Teacher’s Report Form and 1991 Profile. Burlington, VT: University of Vermont, Department of Psychiatry (1991).

[61] Dishion TJ Patterson GR Stoolmiller M Skinner MS. Family, school, and behavioral antecedents to early adolescent involvement with antisocial peers. Dev Psychol. (1991) 27:172–80. 10.1037/0012-1649.27.1.172

[62] Yochim BP Baldo JV Kane KD Delis DC. D-KEFS tower test performance in patients with lateral prefrontal cortex lesions: the importance of error monitoring. J Clin Exp Neuropsychol. (2009) 31:658–63. 10.1080/13803390802448669 19031323

[63] Eglit GM Jurick SM Delis DC Filoteo JV Bondi MW Jak AJ. Utility of the D-KEFS color word interference test as an embedded measure of performance validity. Clin Neuropsychol. (2020) 34:332–52. 10.1080/13854046.2019.1643923 31352854

[64] Satterthwaite TD Wolf DH Erus G Ruparel K Elliott MA Gennatas ED Functional maturation of the executive system during adolescence. J Neurosci. (2013) 33:16249–61. 10.1523/JNEUROSCI.2345-13.2013 24107956 PMC3792462

[65] Shanmugan S Wolf DH Calkins ME Moore TM Ruparel K Hopson RD Common and dissociable mechanisms of executive system dysfunction across psychiatric disorders in youth. Am J Psychiatry. (2016) 173:517–26. 10.1176/appi.ajp.2015.15060725 26806874 PMC4886342

[66] Muthén LK Muthén BO. Mplus User’s Guide. 8th edn. Los Angeles, CA: Muthén & Muthén (1998–2017).

[67] Hair JF Babin BJ Anderson RE Black WC. Multivariate Data Analysis. 8th ed. Andover, United Kingdom: Cengage (2018).

[68] Bauer DJ Hussong AM. Psychometric approaches for developing commensurate measures across independent studies: traditional and new models. Psychol Methods. (2009) 14:101–25. 10.1037/a0015583 19485624 PMC2780030

[69] Bauer DJ. A more general model for testing measurement invariance and differential item functioning. Psychol Methods. (2017) 22:507–26. 10.1037/met0000077 27266798 PMC5140785

[70] Rutkowski L Svetina D. Assessing the hypothesis of measurement invariance in the context of large-scale international surveys. Educ Psychol Meas. (2014) 74:31–57. 10.1177/0013164413498257

[71] DeLong ER DeLong DM Clarke-Pearson DL. Comparing the areas under two or more correlated receiver operating characteristic curves: a nonparametric approach. Biometrics. (1988) 44:837–45. 10.2307/2531595 3203132

[72] Benjamini Y Hochberg Y. Controlling the false discovery rate: a practical and powerful approach to multiple testing. J R Stat Soc Ser B. (1995) 57:289–300. 10.1111/j.2517-6161.1995.tb02031.x

[73] Satorra A Bentler PM. Ensuring positiveness of the scaled difference chi-square test statistic. Psychometrics. (2010) 75:243–8. 10.1007/s11336-009-9135-y PMC2905175 20640194

[74] Boorsboom D. Commentary: when does measurement invariance matter? Med. Care. (2006) 44:S176–81. 10.1097/01.mlr.0000245143.08679.cc 17060825

[75] Teresi JA Ramirez M Jones RN Choi S Crane PK. Modifying measures based on differential item functioning (DIF) impact analyses. J Aging Health. (2012) 24:1044–76. 10.1177/0898264312436877 22422759 PMC4030595

[76] Teresi JA Wang C Kleinman M Jones RN Weiss DJ. Differential item functioning analyses of the patient-reported outcomes measurement information system (PROMIS®) measures: methods, challenges, advances, and future directions. Psychometrika. (2021) 86:674–711. 10.1007/s11336-021-09775-0 34251615 PMC8889890

[77] Ellis RA Bailey AJ Jordan C Shapiro H Greenfield SF McHugh RK. Gender differences in illicit drug access, use and use disorder: analysis of national survey on drug use and health data. J Psychiatr Res. (2024) 175:118–22. 10.1016/j.jpsychires.2024.05.017 38728914 PMC11374475

[78] McHugh RK Votaw VR Sugarman DE Greenfield SF. Sex and gender differences in substance use disorders. Clin Psychol Rev. (2018) 66:12–23. 10.1016/j.cpr.2017.10.012 29174306 PMC5945349

[79] Volkow ND Blanco C. Substance use disorders: a comprehensive update of classification, epidemiology, neurobiology, clinical aspects, treatment and prevention. World Psychiatry. (2023) 22:203–29. 10.1002/wps.21073 37159360 PMC10168177

[80] Castellano-García F Benito A Jovani A Fuertes-Sáiz A Marí-Sanmillán MI Haro G. Sex differences in substance use, prevalence, pharmacological therapy, and mental health in adolescents with attention-deficit/hyperactivity disorder (ADHD). Brain Sci. (2022) 12:590. 10.3390/brainsci12050590 35624977 PMC9139081

[81] Ahmed L de Fockert JW. Focusing on attention: the effects of working memory capacity and load on selective attention. PLoS One. (2012) 7:e43101. 10.1371/journal.pone.0043101 22952636 PMC3429456

[82] Curci A Lanciano T Soleti E Rimé B. Negative emotional experiences arouse rumination and affect working memory capacity. Emotion. (2013) 13:867. 10.1037/a0032492 23731432

[83] Kofler MJ Rapport MD Bolden J Sarver DE Raiker JS Alderson RM. Working memory deficits and social problems in children with ADHD. J Abnorm Child Psychol. (2011) 39:805–17. 10.1007/s10802-011-9492-8 21468668

[84] Fishbein DH Dariotis JK. Personalizing and optimizing preventive intervention models via a translational neuroscience framework. Prev Sci. (2019) 20:10–20. 10.1007/s11121-017-0851-8 29101644

[85] Whelan R Watts R Orr CA Althoff RR Artiges E Banaschewski T Neuropsychosocial profiles of current and future adolescent alcohol misusers. Nature. (2014) 512:185–9. 10.1038/nature13402 25043041 PMC4486207

[86] Conrod PJ O’Leary-Barrett M Newton N Topper L Castellanos-Ryan N Mackie C Effectiveness of a selective, personality-targeted prevention program for adolescent alcohol use and misuse: a cluster randomized controlled trial. JAMA Psychiatry. (2013) 70:334–42. 10.1001/jamapsychiatry.2013.651 23344135

[87] Riggs NR Greenberg MT Kusché CA Pentz MA. The mediational role of neurocognition in the behavioral outcomes of a social-emotional prevention program in elementary school students: effects of the PATHS curriculum. Prev Sci. (2006) 7:91–102. 10.1007/s11121-005-0022-1 16572300

[88] Miller S Boxmeyer C Romero D Powell N Jones S Lochman J. Theoretical model of mindful coping power: optimizing a cognitive behavioral program for high-risk children and their parents by integrating mindfulness. Clin Child Fam Psychol Rev. (2020) 23:393–406. 10.1007/s10567-020-00312-6 32086629

[89] Muratori P Bertacchi I Giuli C Lombardi L Bonetti S Nocentini A First adaptation of coping power program as a classroom-based prevention intervention on aggressive behaviors among elementary school children. Prev Sci. (2015) 16:432–9. 10.1007/s11121-014-0501-3 24942813

[90] Van Ryzin MJ Dishion TJ. The impact of a family-centered intervention on the ecology of adolescent antisocial behavior: modeling developmental sequelae and trajectories during adolescence. Dev Psychopathol. (2012) 24:1139–55. 10.1017/S0954579412000582 22781876 PMC3395237

[91] Hecht ML Shin Y Pettigrew J Miller-Day M Krieger JL. Designed cultural adaptation and delivery quality in rural substance use prevention: an effectiveness trial for the keepin’it REAL curriculum. Prev Sci. (2018) 19:1008–18. 10.1007/s11121-018-0937-y 30056616 PMC6202132

[92] Motamedi M Caldwell L Wegner L Smith E Jones D. Girls just want to know where to have fun: preventing substance use initiation in an under-resourced community in South Africa through HealthWise. Prev Sci. (2016) 17:700–9. 10.1007/s11121-016-0654-3 27129478 PMC4969046

[93] Smith EA Palen LA Caldwell LL Flisher AJ Graham JW Mathews C Substance use and sexual risk prevention in Cape Town, South Africa: an evaluation of the HealthWise program. Prev Sci. (2008) 9:311–21. 10.1007/s11121-008-0103-z 18836890

[94] Ragland JD Turetsky BI Gur RC Gunning-Dixon F Turner T Schroeder L Working memory for complex figures: an fMRI comparison of letter and fractal n-back tasks. Neuropsychology. (2002) 16(3):370–9. 10.1037/0894-4105.16.3.370 12146684 PMC4332798

